# Annual Meetings of Hospitals

**Published:** 1921-05-07

**Authors:** 


### ANNUAL MEETINGS OF HOSPITALS.

### Charing Cross Hospital.

Mr. George Verity, J.P., presiding at the annual meeting on April 29, said that the scheme of voluntary payments from patients has proved a great success. When matters are more settled a full meeting of the Governors will be held under the presidency, he hopes, of Princess Louise, to discuss the threatened alteration in the working of voluntary hospitals. Chiefly owing to the fight put up by Charing Cross Hospital, the attack on the voluntary system has so far failed. There has been some loss of income, but owing to the extraordinary loyalty of subscribers they are able to bring forward a balance in hand of £4,580. The report, which, on the motion of Mr. Verity, seconded by the Rev. S. W. Oxford, M.D., was adopted, states that essential alterations and repairs carried out during the year cost £1,931. The total expenditure was £55,743. The increase of £5,000, as compared with 1919, is partly attributable to a natural increase in prices, and partly to the increased number of patients, the beds occupied daily having been 217, as against 183 in 1919. The cost per occupied bed has decreased from £4 7s. 11d. per week to £3 17s. 4d.—proof that a full hospital can be worked more cheaply than one partially occupied.

### West London Hospital.

The Bishop of Kensington presided at the annual meeting. The report records the gratifying fact that the total income for the year amounted to £60,145, and exceeded the expenditure by £16,146. The grants from the King's Fund and the National Relief Fund contributed to this, and workmen in the districts evinced their gratitude and appreciation of the services rendered by the institution by raising £2,520, an amount nearly £800 in excess of previous contributions. During the year £4,800 was collected in small sums in boxes. Entertainments were instrumental in adding £1,589 to the hospitals funds, and £4,312 was received from the Ministry of Health for the venereal department. The average cost per in-patient per week has risen to £3 16s. 7d., an increase of 9s. 11d. Each out-patient attendance cost the institution 1s. 4d. It has been decided to impose a charge of £1 1s. a week for adult in-patients; for children from seven to fifteen, 14s.; and for those under seven, 7s. a week. Out-patients will pay 6d. an attendance and an additional 6d. for each attendance in the x-ray, electrical, dental, and massage departments. Any patient unable to pay will be treated free. A systematic collection organised by the Education Department of the L.C.C. resulted in nearly £1,000 being received by the hospital, and, in the opinion of the Committee, constitutes a strong argument for the retention of the voluntary principle. The plans for the new out-patient department have now been approved by the King's Fund. Mr. Dan Mason has most generously undertaken to provide £22,000 towards the cost. The King's Fund has allocated £8,000 from the surplus Red Cross funds, and it is hoped that before the next annual meeting the building will be completed.

### Royal Eye Hospital.

Lord Southwark presided at the annual meeting; 781 in-patients were admitted during the year, exceeding those of the previous year by 78. The average number of days each patient was resident was sixteen, and 82 per cent. of the beds were constantly occupied during the year. Since January 1, 1920, out-patients, except old age pensioners, L.C.C. patients, children, and very poor persons, had paid 6d. at each attendance. Payments received under that head totalled £1,526. The question of payment by in-patients is being considered by the Council.

